# Transcriptional and Proteomic Profiling of *Aspergillus flavipes* in Response to Sulfur Starvation

**DOI:** 10.1371/journal.pone.0144304

**Published:** 2015-12-03

**Authors:** Ashraf S. A. El-Sayed, Marwa A. Yassin, Gul Shad Ali

**Affiliations:** 1 Botany and Microbiology Department, Faculty of Science, Zagazig University, 44519, Zagazig, Egypt; 2 Mid-Florida Research and Education Center, Department of Plant Pathology, University of Florida, Apopka, Florida 32703, United States of America; Murdoch University, AUSTRALIA

## Abstract

*Aspergillus flavipes* has received considerable interest due to its potential to produce therapeutic enzymes involved in sulfur amino acid metabolism. In natural habitats, *A*. *flavipes* survives under sulfur limitations by mobilizing endogenous and exogenous sulfur to operate diverse cellular processes. Sulfur limitation affects virulence and pathogenicity, and modulates proteome of sulfur assimilating enzymes of several fungi. However, there are no previous reports aimed at exploring effects of sulfur limitation on the regulation of *A*. *flavipes* sulfur metabolism enzymes at the transcriptional, post-transcriptional and proteomic levels. In this report, we show that sulfur limitation affects morphological and physiological responses of *A*. *flavipes*. Transcription and enzymatic activities of several key sulfur metabolism genes, ATP-sulfurylase, sulfite reductase, methionine permease, cysteine synthase, cystathionine β- and γ-lyase, glutathione reductase and glutathione peroxidase were increased under sulfur starvation conditions. A 50 kDa protein band was strongly induced by sulfur starvation, and the proteomic analyses of this protein band using LC-MS/MS revealed similarity to many proteins involved in the sulfur metabolism pathway.

## Introduction

*Aspergillus flavipes* is a nutritionally facultative fungus, widely distributed in the rhizosphere [1] and as an endophyte in various plants [2]. *A*. *flavipes* has recently been explored for the production of sulfur amino acid metabolizing enzymes such as L-methionine γ-lyase, homocysteine γ-lyase, cystathionine γ-lyase, and glutathione-homocystine oxidoreductase [3–8]. These enzymes exhibit a remarkable pharmaceutical potential for use against cardiovascular diseases and cancer. Endophytic isolates of *A*. *flavipes* from marine plants were shown to produce secondary metabolites including spiroquinazolines, cerebrosides, isobenzofurans, cytochalasins and butyrolactones with broad range antimicrobial, cytotoxic and antiviral activities [1, 9–12]. Gene expression and metabolomic activities of *A*. *flavipes* are affected by the availability of nutrients, especially exogenous sulfur, which is directly incorporated into L-methionine, required for many essential metabolic processes [7, 13]. Metabolic adaptability based on nutrient availability is increasingly reported as a major modulator of physiological behavior of this fungus. However, detailed metabolic status of *A*. *flavipes* under sulfur limitation has not been fully described. In this study, we addressed enzymatic, proteomic and transcriptomic responses of this fungus under sulfur limitation conditions.

In filamentous fungi, sulfur containing amino acids, methionine and cysteine, as well as inorganic sulfur are the most metabolized sulfur sources via the methionine-cysteine cycle [14]. Sulfur uptake, a key step in sulfur assimilation, is mediated by plasma membrane sulfate permease [15]. This enzyme is highly regulated by sulfur repression (metabolite repression). In *Aspergillus* and *Penicillium*, this enzyme is encoded by two genes *sutA* and *sutB* (sulfate transporter) of SulP family [15–17]. In *Neurospora crassa*, sulfate permease I and II are encoded by *cys-13* and *cys-14*, respectively [18, 19]. Transcription of the sulfate permease gene of fungi is strongly regulated by sulfur levels and is repressed by methionine supplementation in the medium [20]. Transcriptional regulation of sulfur metabolism is dependent on the Sulfur Metabolite Repression (SMR) system that consists of the *metR* gene encoding a bZIP transcriptional factor, which controls the expression of all sulfur metabolizing enzymes [14, 21, 22]. In *A*. *nidulans*, the SMR system is controlled by four sulfur controller genes *scon A*, *B*, *C* and *D*, since mutations in these genes cause loss of SMR gene expression [22, 23]. Once transported to the cytosol, sulfate is first activated by ATP sulfurylase to adenosine 5'-phosphosulfate (APS), then converted to 3'-phosphoadenosine 5'-phosphosulfate (PAPS) by APS-kinase, and then PAPS is reduced to sulfite by PAPS reductase [14]. The sulfite molecule is further reduced to sulfide by sulfite reductase [21]. Subsequently, sulfide is incorporated with *O*-acetylserine or *O*-acetyl-homoserine to form cysteine and homocysteine by cysteine synthase and homocysteine synthase, respectively [19]. Simultaneously, cysteine and homocysteine undergo transsulfuration and reverse transsulfuration forming L-methionine, glutathione and polyamines [4].

Sulfur assimilation and metabolism have been extensively studied in filamentous fungi such as *A*. *nidulans* [24], *Neurospora crassa* [19], and yeasts, *Yarrowia lipolytica* [25], *Saccharomyces cerevisiae* [21] and *Schizosaccharomyces pombe* [26]. It plays an important role in the pathogenicity and virulence of *A*. *fumigatus* [27]. From a pharmaceutical perspective, *A*. *flavipes* has great potential for the production of sulfur amino acid metabolizing enzymes [4] and volatile sulfur compounds [3–8]. However, there are no reports describing the kinetics of sulfate assimilation and metabolism of sulfur amino acids in *A*. *flavipes*.

The objective of this work was to study the molecular expression and proteomic profiling of *A*. *flavipes* sulfur metabolizing enzymes in response to nutritional sulfur limitation. The activity and expression of *A*. *flavipes* enzymatic system controlling sulfur transport, assimilation and metabolism of sulfur amino acids were assessed under sulfur starved growth conditions. Proteomic analysis using LC-MS/MS was conducted to identify prominently induced proteins in response to sulfur limitation. Relationships between gene expression and enzymatic activities of several key enzymes that were investigated in this report are discussed in the context of *S* availability and limitation.

## Materials and Methods

### *Aspergillus flavipes* strain and growth conditions

*Aspergillus flavipes* (Bainier et Sartory), anamorph (ATCC 24487) was maintained on PDA and Dox's medium [28]. The fungus was grown on basal minimal medium containing 1% glucose, 70 mM NaNO_3_, 7 mM KCl, 11 mM K_2_HPO_4_, 0.25 mM MgSO_4_.7H_2_O and 1.5% agar [27]. For sulfur starvation and resupply experiments, MgSO_4_ was replaced with MgCl_2_ in the Dox’s medium. Sulfur starvation experiments were performed as follows: One mL spore suspension (10^8^ spore•ml^-1^) prepared from a 5-day old *A*. *flavipes* culture on PDA was added to sulfur free basal medium [27], and incubated at 30°C for 24 h with shaking at 120 rpm. One ml of this culture was inoculated into solid and liquid media supplemented with either L-methionine (5 mM), L-cysteine (5 mM), L-cystine (5mM), MgSO_4._7 H_2_O (2 mM) or glutathione (5 mM). Amino acids were filter-sterilized (0.22 μm Millipore) and then added to the basal minimal medium at desired concentrations. Cultures were incubated at the same conditions as above up to 6 days. Fungal tissues were collected by filtration, washed with sterile potassium phosphate buffer (pH 7.5) and kept at -20°C for subsequent physiological and proteomic analyses.

### Measurement of fungal morphology, growth kinetics, sulfur uptake and glutathione pool

The effect of sulfur starvation on morphological growth of *A*. *flavipes* was determined by growing the fungus on Dox’s medium amended with sulfur sources as mentioned above. The fungus was incubated at 30°C and the rate of radial growth as well as colony morphology of the fungus was photographed with a digital camera (Canon, USA). After culturing of *A*. *flavipes*, the residual concentration of L-methionine and cysteine was determined as described before [7, 29]. Total glutathione concentration of *A*. *flavipes* was determined using Ellman’s reagent [5,5'-Dithio-*bis*-(2-nitrobenzoic acid)] [30, 31]. Briefly, the collected mycelial pellets (1.0 gm) were washed two times with sterile saline solution (0.8% NaCl), ground in liquid nitrogen, vigorously mixed in cold 5% 5-sulfosalicylic acid and incubated on ice for 30 min. The mixture was centrifuged at 5000xg for 15 min at RT and the supernatant was transferred to new tubes and neutralized with triethanolamine. Total glutathione (GSH and GSSG) of the supernatant was measured using the DTNB assay [32]. For measuring GSH only, the mycelium was treated with *N*-ethylmaleimide (NEM) to prevent autooxidation of GSH to GSSG [32].

### Assessment of sulfur metabolizing enzymes activity and intracellular protein

For assaying total intracellular protein and activity of sulfur-metabolizing enzymes, mycelial pellets were collected and washed with 100 mM potassium phosphate (PP) buffer, pH 7.5. The mycelial pellet (1gm) was ground in a chilled mortar and thoroughly suspended in PP buffer (5 mL) containing 1 mM EDTA and 1mM PMSF. The homogenate was sonicated for 1 min at 60% amplitude in a sonicator (550 Sonic Dismembrator, Fisher Scientific). The homogenate was centrifuged at 5000xg for 15 min and the supernatant was used as a source of enzymes as describe below [7].

The activity of ATP-sulfurylase was assessed using sodium molybdate assay [33]. Assay contained 0.1 M Tris-HCl (pH 7.5), 5 mM Na_2_MoO_4_, 10 mM MgCl_2,_ 10 mM ATP and 100 μl of enzyme extract in a total volume of l mL. Blank reactions contained 100 μl water instead of sodium molybdate. After incubation for 20 min at 37°C, the reaction was stopped by adding 10% TCA and centrifuged at 5000xg for 10 min. A 100 μl fraction of the reaction was mixed with 200 μl of 2.5% Na_2_MoO_4_ in 2.5 M H_2_SO_4_ and incubated for 10 min at 37°C. The mixture was then incubated for an additional 10 min with 100 μl of Eikonogen solution (0.25% dissolved in 14% metabisulfite), and the developed color was measured at A_660_ nm in a microtiter plate reader (Synergy H1 microplate reader, BioTek, USA).

The activity of sulfite reductase was measured by sulfide assay [34]. Briefly, the assay reaction (1 ml) contained 100 mM Tris-HCl (pH 7), 0.5 mM NADP, 1.5 μM FAD, 5 mM glucose-6-phosphate, 0.5 mM sodium bisulfite and 100 μl of enzyme extract. Blank reactions contained water instead of sodium bisulfite. The reaction was incubated at 37°C for 20 min and stopped by cooling on icebath for 15 min. Immediately after cooling, 2 mM N,N-dimethyl-p-phenylenediamine (in 6.5 N HCl) and 20 mM FeCl_3_ (in 1.2 N HCl) was added to each reaction [35]. After 20 min, the developed methylene blue color was measured at A_670_ nm a microtiter plate reader. Enzyme activity was expressed in units (U), which is defined as 1 μmole of sulfide released per min. Absorbance values (A_670_ nm) were converted to sulfide amounts (μmoles) from the plot of standard curve of a series of sulfide concentrations (μM) against A_670_ nm absorbance.

The activity of cysteine synthase was determined [36]. Each reaction contained 100 mM potassium phosphate (pH 7.8), 5 mM *O*-acetylserine, 3 mM sodium sulfide, 10 mM DTT and 100 μl of enzyme extract in 1 ml total reaction volume. After incubation at 37°C for 20 minutes, the reaction was stopped by adding 500 μl acidic Ninhydrin reagent (1% ninhydrin in HCl: glacial acetic acid, 1:4 (v/v)) [37]. The mixture was boiled for 5 min and the developed color was measured at 550 nm. The activity unit was defined as the amount of enzyme releasing 1 μmole of cysteine per min, determined from the curve of absorbance against cysteine standards.

The activity of methionine γ-lyase and cystathionine β, γ-lyases were determined using 5,5’-dithiobis (2-nitrobenzoic acid) (DTNB) reagent [7, 8]. Glutathione reductase was assayed based on NADH oxidation [31]. Each reaction contained 1 μM FAD, 0.1 mM NADH, and 10 mM oxidized glutathione (GSSG) and 100 μl of the enzyme extract in 1 ml total volume. After 10 min incubation at 30°C, the decrease of NADH concentration was measured at 340 nm using a microtiter plate reader.

Glutathione peroxidase activity was based on measuring the residual concentration of glutathione (GSH) using the DTNB assay [38]. Each reaction contained 1 mM GSH, 1 mM H_2_O_2_ and 100 μl enzyme extract in potassium phosphate buffer (pH 7.0) as described above. After incubation at 30°C for 10 minutes, residual GSH was determined. The activity of glutathione peroxidase was determined from the residual GSH concentrations, since no chemical reaction occurs between DTNB reagent and GSSG [38].

The concentration of total intracellular protein was assessed by Bradford assay (Bio-Rad Assay Kit, cat#500–0006) using bovine serum albumin as standard.

### Reverse Transcription quantitative (RT-qPCR) analyses of sulfur metabolizing enzyme genes, and the *metR* and *scon* genes

Pivotal enzymes implicated in sulfur assimilation and sulfur amino acid metabolism are sulfate permease, ATP-sulfurylase, sulfite reductase, APS-kinase, APS-reductase, arylsulfatase, methionine permease, cystathionine β, γ-lyase, methioninase, cysteine synthase, S-adenosylhomocysteinase, homocysteine synthase, glutathione reductase and glutathione peroxidase [14]. Differential expression of these genes as well as those of the sulfur transcriptional activator *metR* and sulfur controller *scon* genes [14, 22] in response to sulfur starvation was determined using qPCR. The primer sequences for qPCR analysis of these genes are listed in Table 1. The *A*. *flavipes* actin gene, *actaA*, was used as internal standard for normalizing total amount of RNA between samples.

**Table 1:** List of Primers for Real-Time PCR analysis.

Enzyme	Gene ID	Primers (F;R)
Sulfate Permease	KF483582.1	5'- GAGCCGGTCTATCTTCTTGC- 3'; 5'- TCGGTGTAGTGATTGGCATT- 3'
APS-Kinase	XM001825217	5'- CAAGTCTACCATTGCCGTTG- 3'; 5'- ACCGAGGTCCTTGTTGAGTC- 3'
Arylsulfatase	XM743459.1	5'- ACCCTTTCTTCCAAACAACG- 3'; 5'- TAGGACGCTCGGAAAGAAAT- 3'
ATP-Sulfurylase	XM002383898.1	5'- TGAGATCAAGGGCTTCACTG- 3'; 5'- ACATCGACAGTGAGGTGAGC- 3'
Cysteine Synthase	XM742230.1	5'- AACTTCGAAGCAGGAAAGGA- 3';5'- GCACTGCTACTTCCAACGAA- 3'
Homocysteine Synthase	XM742825.1	5'- TAAGCACGCTGACAGATTCC- 3'; 5'- AAGGATCTCAACCCGAACAC- 3'
S-Adenosylmethionine Synthase	XM001825239.2	5'- GAGGGATGTTACGGCGTTAT- 3'; 5'- TTGTCCGAAGCACAGCTTAC- 3'
METR *A*. *fumigatus* Af293 bZIP	XM747080.1	5'- TCAACCTCGATGCTGAACTC- 3'; 5'- CCGTTGTCTTTGCACTGTCT- 3'
METR *A*. *oryzae*	XM001821377.2	5'- GTGGAGAGCGACAGAATGAA- 3'; 5'- CTGAATAATCGGGCATGTTG- 3'
*ActA A*. *fumigatus* Af293	XM749985.1	5'- GACTGGTTTGGCAATTGATG- 3'; 5'- GCATCAGTGATCTCACGCTT- 3'
(scon-2) gene	XM957732.2	5'- ACAAGGGAGGGTCACAGAAC- 3'; 5'- GCTTTCCATGTTGATTGACG- 3'
Methionine Permease	XM001817382.2	5'- TCCTATCTCGTTTCGCCAATCTTC- 3'; 5'-TCGCACATCGATAGTGACAAGATG-3'
Cystathionine γ-Lyase	XM742148.1	5'-ATGACTGCATCTTCCAACGGTCACG-3';5'-TCCGTTCTCCAACTGCCTGGCTAGCGA-3'

*A*. *flavipes* was cultured on the desired S-starving conditions as mentioned above for 24 h. After incubation of the liquid fugal cultures under control and *S*-starvation conditions, the mycelial fungal pellets were collected by centrifugation at 5000xg, washed with sterile 100 mM potassium phosphate buffer (pH 7.0) and stored at -80°C until further use. Total RNA was isolated from these samples using the RNeasy Plant Mini Kit (QIAGEN, USA). The concentration and purity of RNA was assessed by NanoDrop (Thermo Scientific) and running on 1% agarose gel. Total RNA (1 μg) was first treated with RNase-free DNAse I (Fermentas), and then reverse transcribed using the SuperScript III First Strand Synthesis Kit (Invitrogen) according to the manufacturer instructions. The first strand cDNA was then used as template in qPCR reactions using iQ^TM^ SYBR Green Supermix (Bio-Rad). qPCR reactions were performed in a real time PCR machine (Light Cycler 480, Roche, USA) using the following thermal profile: Initial denaturation at 95°C for 3 min, followed by 50 cycles of 95°C for 15 s, 55°C for 30 s (annealing), 72°C for 1 min (extension). Melting curve analyses were performed at 55–95°C. Each sample was run in triplicate. Data were normalized using the constitutively expressed actin-encoding gene (*actaA*) of *A*. *flavipes* as endogenous control. Relative fold change of the target genes was calculated from the standard curve of relative quantification (Bookout et al., 2006). Statistical comparisons were conducted using the Student’s *t*-test and *p*-value ≤ 0.05 were considered significant. Data are presented as fold change between the *S*-starved and non-starved fungal cultures.

### SDS-PAGE protein profiling

Total protein from each sample at the tested nutritional conditions was extracted by grinding fungal tissues in a chilled mortar followed by suspension in protein extraction PP buffer (100 mM, pH 7.5) containing 1 mM EDTA, 1% 2-mercaptoethanol and 1mM PMSF [7]. The intracellular protein was extracted from the sulfur starved cultures grown on various sulfur sources, in addition to the positive controls (non-starved) and negative controls (grown on sulfur free medium). The total extracted protein was electrophoresed by gradient SDS-PAGE (Criterion^TM^, 4–20% Tris-HCl, Bio-Rad), and stained with Coomassie Brilliant Blue (Bio-Rad) [39].

### Proteomic analysis

Effect of sulfur starvation on the kinetics of *A*. *flavipes* proteome was analyzed using Liquid Chromatography-Tandem Mass Spectrometry nanospray ionization (LC-MS/MS) at the Biomolecular and Proteomics Mass Spectrometry Facility, University of California, San Diego, UCSD, USA. Spores of *A*. *flavipes* were first starved for different incubation periods (6, 12, 24, 36, 48 hr), and then added to media amended with 5mM L-methionine as described above. These cultures were incubated for 5 days in a growth chamber at 30°C with continuous shaking at 130 rpm. Total intracellular proteins were extracted as above, and electrophoresed on a gradient SDS-PAG, as described above. Gels were stained with coomassie blue, and a protein band, which was prominently over-induced in response to sulfur starvation, was excised and in-gel digested with trypsin according to a published protocol [40]. After trypsin digestion, dried peptides were dissolved in 20 μl of trifluoroacetic acid prior to proteomic analysis [40]. LC-MS/MS analysis were conducted on the AB SCIEX TripleTOF^TM^ 5600 system fitted with a Nanospray source, and coupled with Tempo nano-flow HPLC using 5-μm C18 Zorbax bead column (10cm x 100μm) (Agilent Technology) [40]. A linear gradient of acetonitrile (ACN) buffer (5.0–60%) with flow rate 250 μl min^-1^ was used for eluting peptides from the column into the mass spectrometer. The ACN gradient buffer was made by mixing buffer A (ACN 2%, formic acid 0.2%, TFA 0.005% in H_2_O) and buffer B (ACN 100%, formic acid 0.2% and TFA 0.005%). MS/MS data were acquired independently in which the MS1 data were acquired for 250 ms at m/z 400–1250, and the MS/MS data were acquired at m/z 50–2000 Da. The raw MS/MS data files were extracted and analyzed using Protein Pilot 4.0 (ABSCIEX) [41] for peptide identification. Since the genome of *A*. *flavipes* is not available, protein identifications were based on the genome of *A*. *fumigatus* [42] (http://www.ncbi.nlm.nih.gov/genome). The identification criteria included at least five peptide fragment ions per protein with E-values < 0.05. Molecular, biological and cellular functions of the identified proteins were annotated and categorized using the Blast2GO (Ver 3.0.10) gene ontology software [43].

## Results

### Morphological and physiological response of *A*. *flavipes* to sulfur starvation

**Fig 1:**
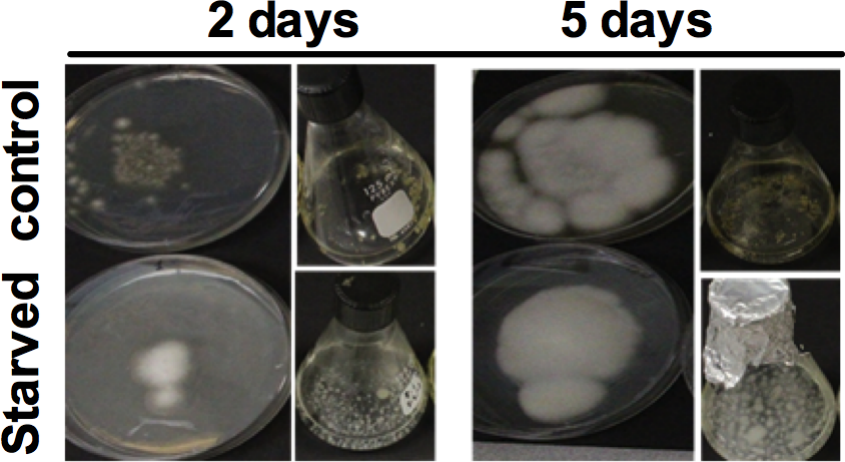
Growth of *A*. *flavipes* in response to non-starved control and sulfur starved conditions. The spores of *A*. *flavipes* were starved of sulfur for 24 h, then inoculated into Dox’s medium amended with MgSO_4_. Sulfur starved fungal spores (Lower) and control (Upper) after 2 days and 5 days. Negative control was cultured on–*S* Dox’s media.

To investigate effect of sulfur (*S*) starvation and re-supply of various *S* sources on morphological and physiological characteristics of *A*. *flavipes*, fungal spores were first grown in sulfur-free liquid medium [27], and then subsequently transferred to media containing various sulfur sources: MgSO_4_, L-Methionine, L-Cysteine, L-Cystine or Glutathione. After 2 days of incubation, non-starved cultures displayed brown pigmentation both on solid media in petri-plates and in liquid media, whereas *S* starved cultures appeared white. On the 5^th^ day of incubation both starved and non-starved had similar morphology and whitish color on solid plates, whereas, in liquid cultures, non-starved controls still displayed brown pigmentation and starved cultures were white (Fig 1).

**Fig 2:**
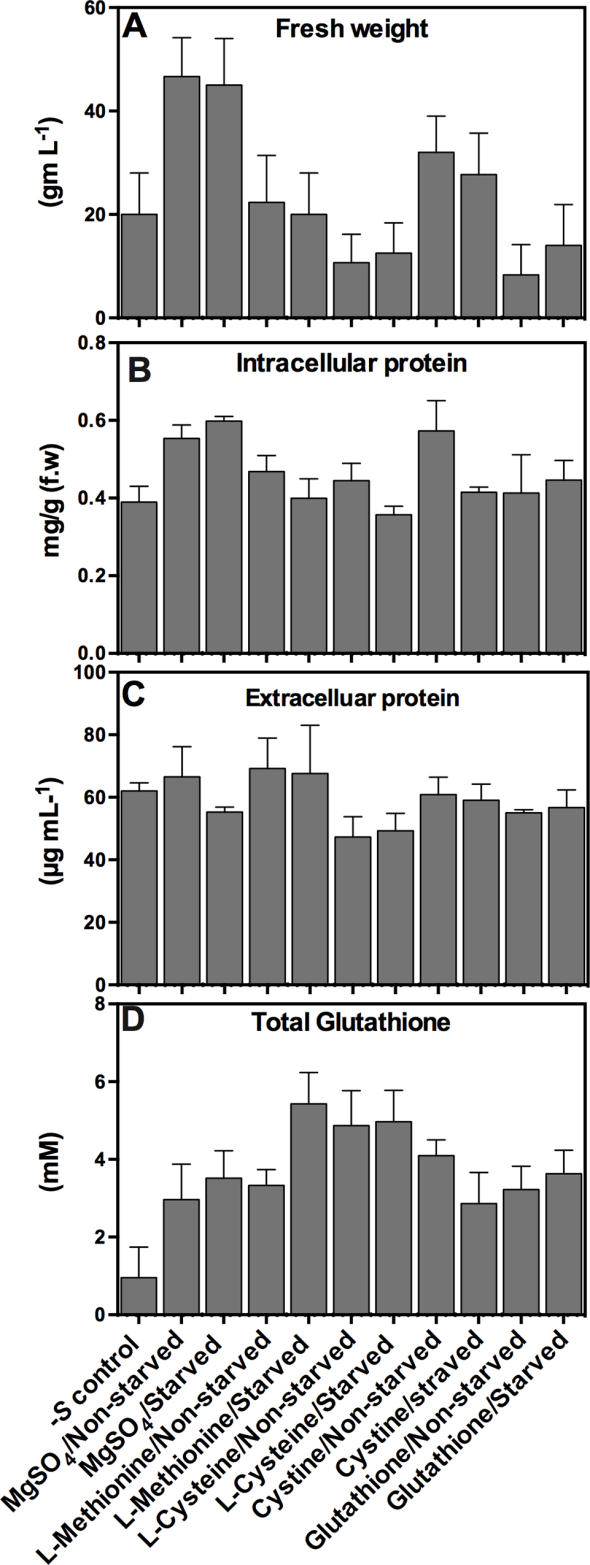
Physiological responses of *A*. *flavipes* to *S* starvation. Spores of *A*. *flavipes* were starved of sulfur (cultured on Dox’s medium with no S compounds) or non-starved control (cultured on Dox’s liquid medium with 5 mM MgSO_4_) for 24 h, then inoculated to Dox’s liquid medium amended with MgSO_4_, L-Methionine, L-Cysteine, L-Cystine and Glutathione.–*S* control represents culture on Dox’s medium with no sulfur compounds. Fresh weight (A) and concentration of total intracellular proteins (B), extracellular proteins (C), and total glutathione (D) were determined after fives day of culture. Data shown are means ± SD of three replications.

Effect of sulfur starvation and of different *S* sources on the physiology of *A*. *flavipes* was assessed by comparing fungal fresh weight, intracellular and extracellular proteins, and total glutathione in starved and non-starved control cultures (Fig 2). Statistical analyses of the data showed that fresh weights were significantly affected by *S* starvation and *S* sources when resupplied in the culture medium. Compared to–*S* control, fresh weight was significantly increased on MgSO_4_ containing media (Fig 2A, *p*<0.001). Unlike the relative higher fresh weights of starved and non-starved cultures of *A*. *flavipes* grown on cystine, all the other *S* sources did not display any significant difference from–*S* control (*p*>0.1). Fresh weights of cultures on all other *S* sources did not display any significant difference from–*S* control (*p*>0.1). Similarly, fresh weights of starved and non-starved spores were also not different when resupplied with any of the tested *S* sources (*P*>0.5). In response to *S* starvation and *S* sources, total intracellular and extracellular proteins of *A*. *flavipes* cultures were variable (Fig 2B and 2C). In comparison to–*S* control, total intracellular protein increased by approximately 30% on media containing MgSO_4_ (for both starved and non-starved spores) and on cystine (only starved spores) (*p*<0.001). A pair-wise comparison of starved versus non-starved control spores on each of *S* sources revealed that only on cystine-containing media, starved cultures had less total intracellular protein than non-starved control. Except for a slight decrease on cysteine-containing media compared to–*S* control, total extracellular protein was not affected by *S* starvation, *S* source or whether spores were starved or not (Fig 2C).

The most dramatic effect of *S* starvation was observed with total glutathione concentration. Total glutathione concentrations increased significantly (3 to 5.7 fold) on all *S* sources compared to–*S* controls. The highest increase was observed with starved spores when resupplied with methionine, and the least increase was observed with starved spores when resupplied with cystine (Fig 2D, *P*<0.001). Pairwise comparison between starved and non-starved cultures revealed that the concentration of glutathione in the *S* starved spores was approximately 1.63x more than in non-starved spores when grown on L-methionine. On the other hand, when grown on L-cystine, glutathione concentration for starved spores was 1.4x less than non-starved spores (Fig 2D).

### Protein profiles of *A*. *flavipes* responding to sulfur starvation conditions

**Fig 3:**
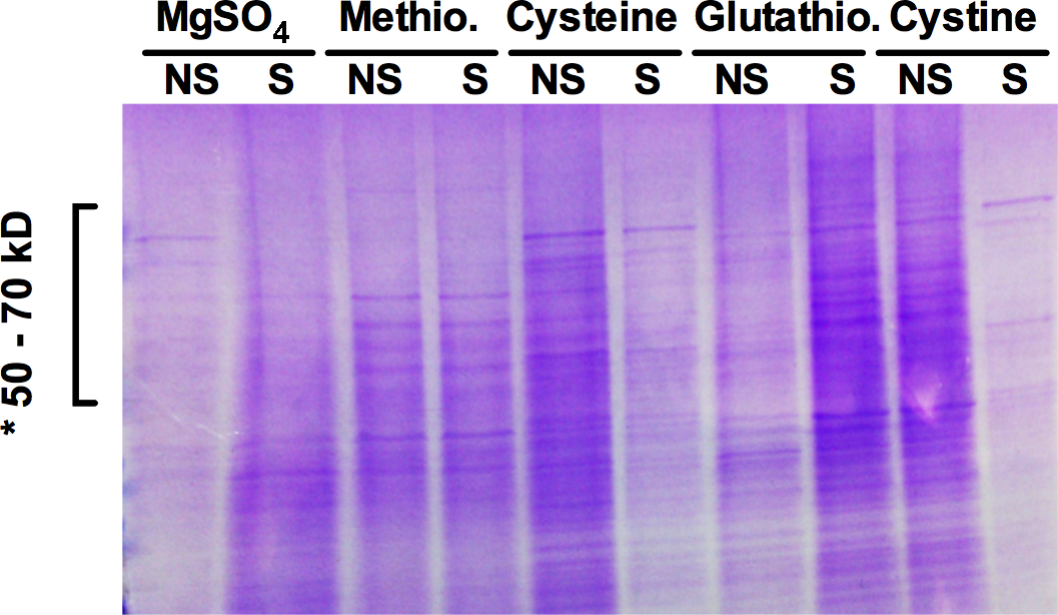
Intracellular protein profile of *A*. *flavipes* in response to sulfur starvation. The sulfur starved (lanes labelled as ‘S’) and non-starved (lanes labelled as ‘NS’) spores of *A*. *flavipes* were inoculated to the basal medium containing the indicated sulfur compounds. Total intracellular protein was run on a gradient SDS-PAGE gel. *, indicates the 50 to 70-kDa region of the gel with prominent changes in the intensity of protein bands.

Effect of *S* starvation and of various *S* sources on differential protein expression was analyzed using SDS-PAGE of total intracellular proteins extracted from 5-day old submerged *A*. *flavipes* cultures on different *S* sources. As shown in Fig 3, substantial changes in protein profiles were observed when cultures were grown on different *S* sources. The most pronounced changes were observed between the 50 and 70 kDa size range, which in general corresponds to the predicted sizes of enzymes involved in *S* metabolism. These observations led us to assess the activity of several key enzymes involved in sulfur uptake and assimilation.

### Activities of the sulfur metabolizing enzymes under sulfur starvation

**Fig 4:**
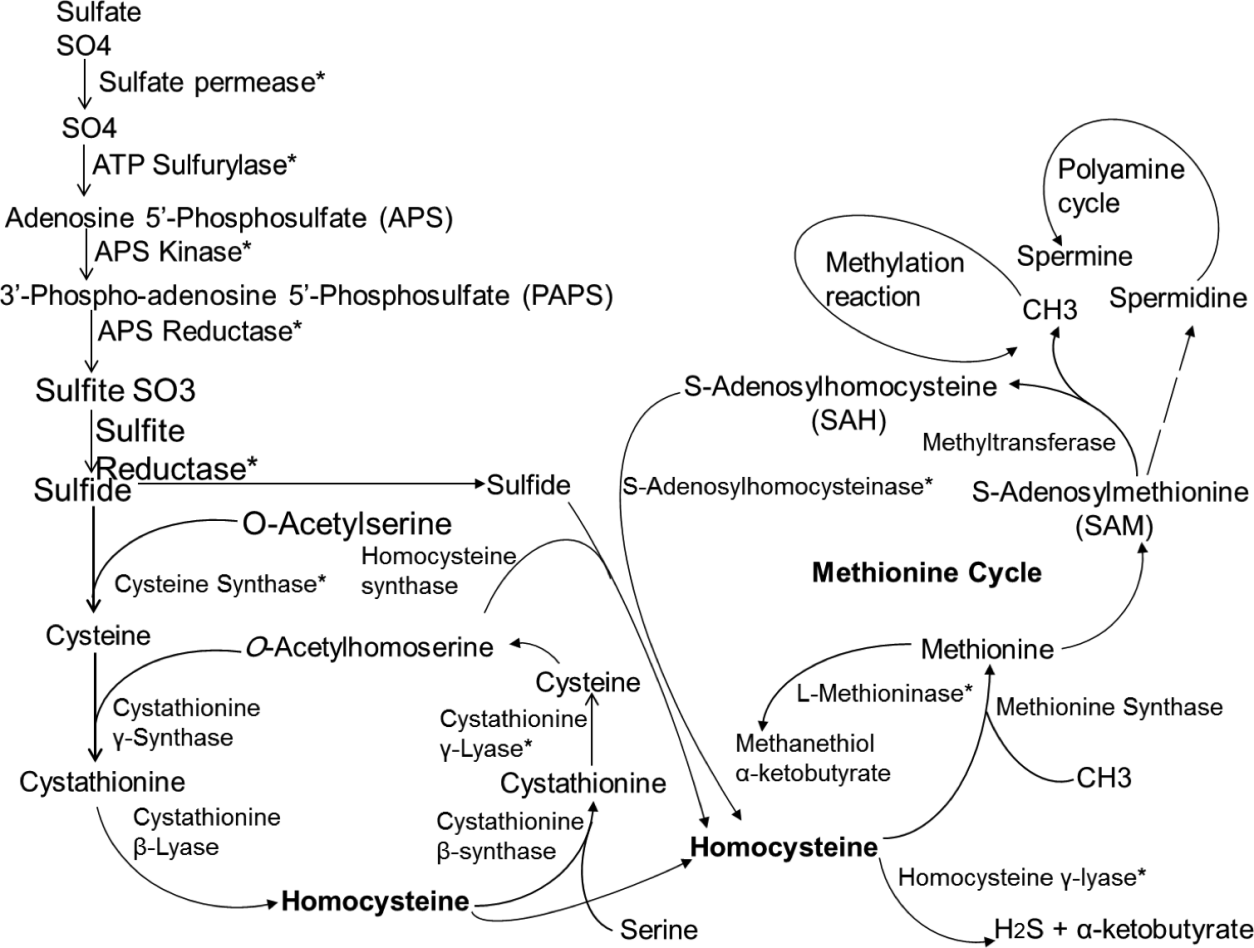
Proposed sulfur metabolism pathway *A*. *flavipes*. This pathway highlights major enzymes involved in the metabolism of sulfur and sulfur amino acids [4].

An overall pathway illustrating position of key enzymes involved in the assimilation of sulfur and sulfur amino acids in fungi is presented in Fig 4 [14, 21, 44]. We hypothesized that some of the differentially expressed protein bands, which fluctuated substantially with different *S* sources (Fig 3) might correspond to these sulfur amino acids metabolizing enzymes. To test this hypothesis, we assayed the activity of several of these key enzymes including ATP-sulfurylase, sulfite reductase, cysteine synthase, cystathionine β-lyase, cystathionine γ-lyase, methionine γ-lyase, glutathione reductase and peroxidase. Spores of *A*. *flavipes* were grown in–*S* or +*S* (MgSO_4_) media for 24 h, then resupplied with various sulfur sources and activities of the above enzymes were assessed as described in the Materials and Methods. In general, all sulfur assimilating enzymes displayed higher activity in response to *S* limitation.

**Fig 5:**
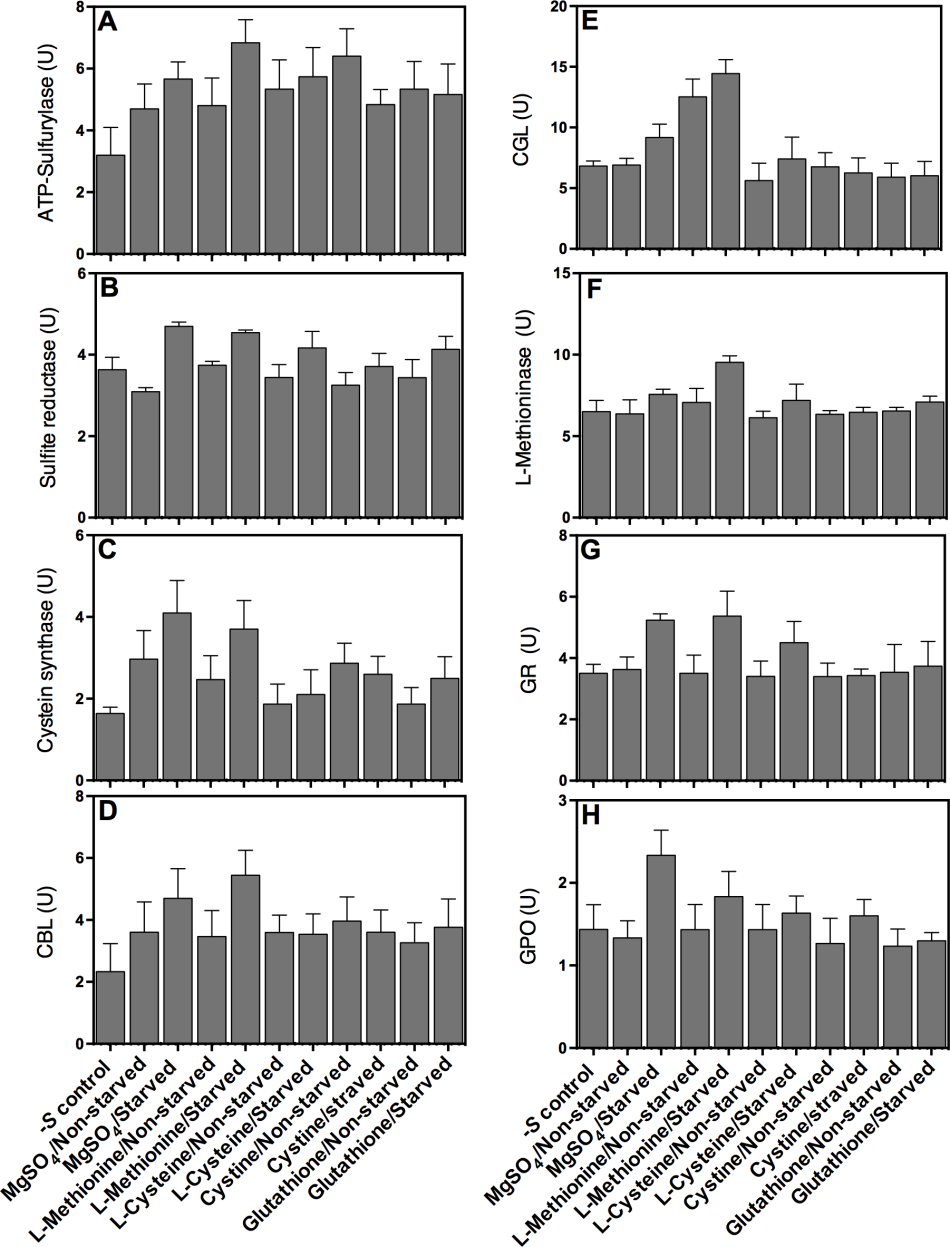
Activity of key *A*. *flavipes* enzymes involved in sulfur and sulfur amino acid metabolism in response to sulfur starvation. Spores of *A*. *flavipes* were starved of sulfur (cultured on Dox’s medium with no S compounds) or non-starved control (cultured on Dox’s liquid medium with 5 mM MgSO_4_) for 24 h, then inoculated to Dox’s liquid medium amended with MgSO_4_, L-Methionine, L-Cysteine and L-Cystine.–*S* control represents culture on Dox’s medium with no sulfur compounds as described in the Methods and Materials section. Enzyme activity of ATP-Sulfurylase (A), sulfite reductase (B), cysteine synthase (C), Cystathionine β- lyase (D), cystathionine γ-lyase (E), L-methioninase (F), Glutathione reductase (G), and glutathione peroxidase (H) were determined as described in Materials and Methods. Data shown are means ± SD of three replications.

Compared to–*S* control, ATP-sulfurylase activity significantly increased on all tested *S* sources (Fig 5A). The highest activity of this enzyme was measured for the starved *A*. *flavipes* grown on L-methionine. Statistical comparison of starved and non-starved treatments revealed that activity of this enzyme in the starved spores increased when transferred to media with L-methionine, decreased with cystine, and remained unchanged for the rest of *S* sources.

Relative to–*S* control, the activity of sulfite reductase (SR) increased for the starved cultures when resupplied with MgSO_4_, L-methionine and L-cysteine; no significant change in SR activity was observed for the remaining treatments. Compared to non-starved spores, SR activity in the *S* starved spores increased significantly (25%, *p*<0.05) when resupplied with MgSO_4,_ L-methionine or L- cystine (Fig 5B).

Cysteine synthase (CS) and cystathionine β-lyase (CBL) followed more or less similar patterns of enzyme activity in response to *S* starvation and to different *S* sources (Fig 5C and 5D). Compared to–*S* control, both enzymes displayed significantly higher activities in the starved spores when resupplied with MgSO_4_ or L-methionine, but not with any of the other *S* sources. Similarly, activities of these two enzymes on MgSO_4_ or L-methionine were also higher (25 to 38%) in starved spores than in non-starved spores. CS activity was 4.0 U/ml (sulfur starved) and 3.2 U/ml (non-starved), when resupplied with MgSO_4_, and 3.5 U/ml (sulfur starved) and 2.2 U/ml (non-starved) with L-methionine. A similar pattern was observed for CBL, which displayed 4.6 U/ml (sulfur starved) and 3.1 U/ml (non-starved) on MgSO_4_, and 5.3 U/ml (sulfur starved) and 3.3 U/ml (non-starved) with L-methionine. The activities of these enzymes for the rest of the treatments were not changed substantially.

Compared to–*S* media, higher CGL activity was observed in the sulfur starved (14.0 U/ml) and non-starved (11.2 U/ml) cultures when grown on media containing L-methionine (Fig 5E). Significantly higher CGL activity was observed in the starved spores compared to non-starved spores when cultured on MgSO_4_. Other than this, the activity of CGL was similar on–*S* media, and for starved and non-starved cultures when grown on L-cysteine, cystine and glutathione. Except significantly higher activity (10 U/ml) in starved spores when grown on L-methioninase, activity was not changed on any of the other treatments (Fig 5F).

Glutathione reductase (GR) and glutathione peroxidase (GPO) displayed more or less similar activity patterns in response to *S* starvation and *S* sources (Fig 5G and 5H). Both these enzymes displayed higher activities in starved spores compared to non-starved spores when grown on media containing MgSO_4_ or L-methionine.

### RT-qPCR analysis of genes encoding sulfur metabolizing enzymes and transcriptional regulators

**Fig 6:**
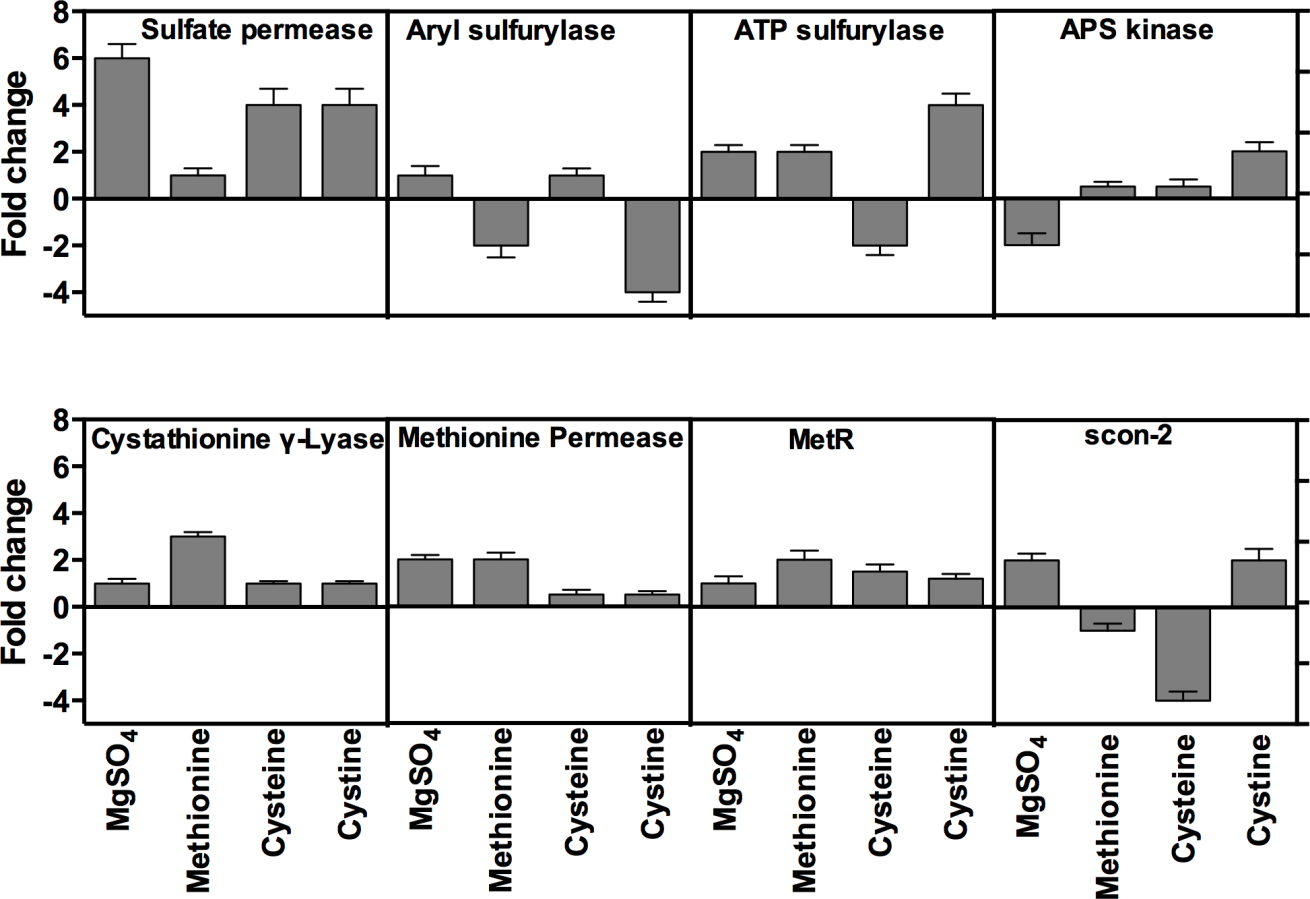
Effect of sulfur starvation on the transcription of sulfur metabolism genes of *A*. *flavipes*. Sulfur starved and non-starved spores of *A*. *flavipes* were transferred to basal Dox’s medium containing MgSO_4_, L-methionine, cysteine, and cystine. Total RNA from the fungal cultures of these treatments was reverse transcribed and used as template in Reverse Transcription (RT)-qPCR using gene-specific primers for the indicated enzymes. Transcription levels were normalized to the transcription of constitutively expressed Actin A gene. Fold changes in transcription of starved cultures were calculated relative to non-starved controls.

To determine if the enzyme activity results described above could be due to higher transcription activity, we analyzed effect of *S* starvation and *S* sources on the transcription of genes encoding these enzymes using Reverse Transcriptase qPCR. *S* starvation and *S* source experiments were performed exactly as described above for assaying the enzyme activities. List of primers used in the qPCR of the analyzed genes is provided in Table 1. qPCR data showed that the transcription of genes encoding sulfate permease, arylsulfatase, ATP-sulfurylase, APS-kinase, methionine permease, cystathionine γ-lyase and the transcriptional regulator genes was changed in response to sulfur starvation. Similar to the enzyme activities profiles, transcriptional profiles also varies substantially on each *S* source, ranging from 6-fold induction to 3-fold repression (Fig 6). In general, transcription of most enzyme genes was increased by *S* starvation; however, there were several notable exceptions. Arylsulfatase was repressed when cultured on cystine (3.5 fold) and L- methionine (2-fold), whereas ATP sulfurylase and APS kinase were repressed on cysteine and MgSO_4_, respectively. In response to *S* starvation, sulfate permease displayed the highest transcriptional induction among all the genes on most of tested *S* sources suggesting that this enzyme plays a key role in overall *S* metabolism. Transcription of CGL in starved spores strongly increased by 3 fold when resupplied with L-methionine, compared to only 1-fold on all other S sources.

Transcription of the sulfur transcriptional regulator gene, *metR*, was induced in *S* starved spores when cultured on all tested sulfur sources with the strongest induction (2 fold) on L-methionine and slight induction (1.2 fold) on MgSO_4_, cysteine and cystine (Fig 6). In contrast, transcription pattern of *scon-2* gene under sulfur limitation conditions was variable; it was induced in starved spores by two folds when cultured on MgSO_4_ and cystine, but down regulated when cultured on L-methionine and cysteine.

### Effect of sulfur starvation on the kinetics of protein expression of *A*. *flavipes*

Enzymatic and transcriptional profiling of *S* metabolism genes as described above suggest that *S* starvation and *S* sources profoundly affect kinetics of gene expression and enzyme activity. We further analyzed protein kinetics in response to *S* starvation using 1D PAGE. We used L-methionine as a standard sulfur source to conduct proteomic analysis of *A*. *flavipes* in response to sulfur limiting conditions. Fungal spores were starved of *S* for 6, 12, 24, 36 and 48 h by growing on–*S* media, then transferred to the basal medium containing L-methionine (5 mM), followed by incubation on a shaker for 5 days. Total intracellular protein from these cultures was profiled by SDS-PAGE.

**Fig 7:**
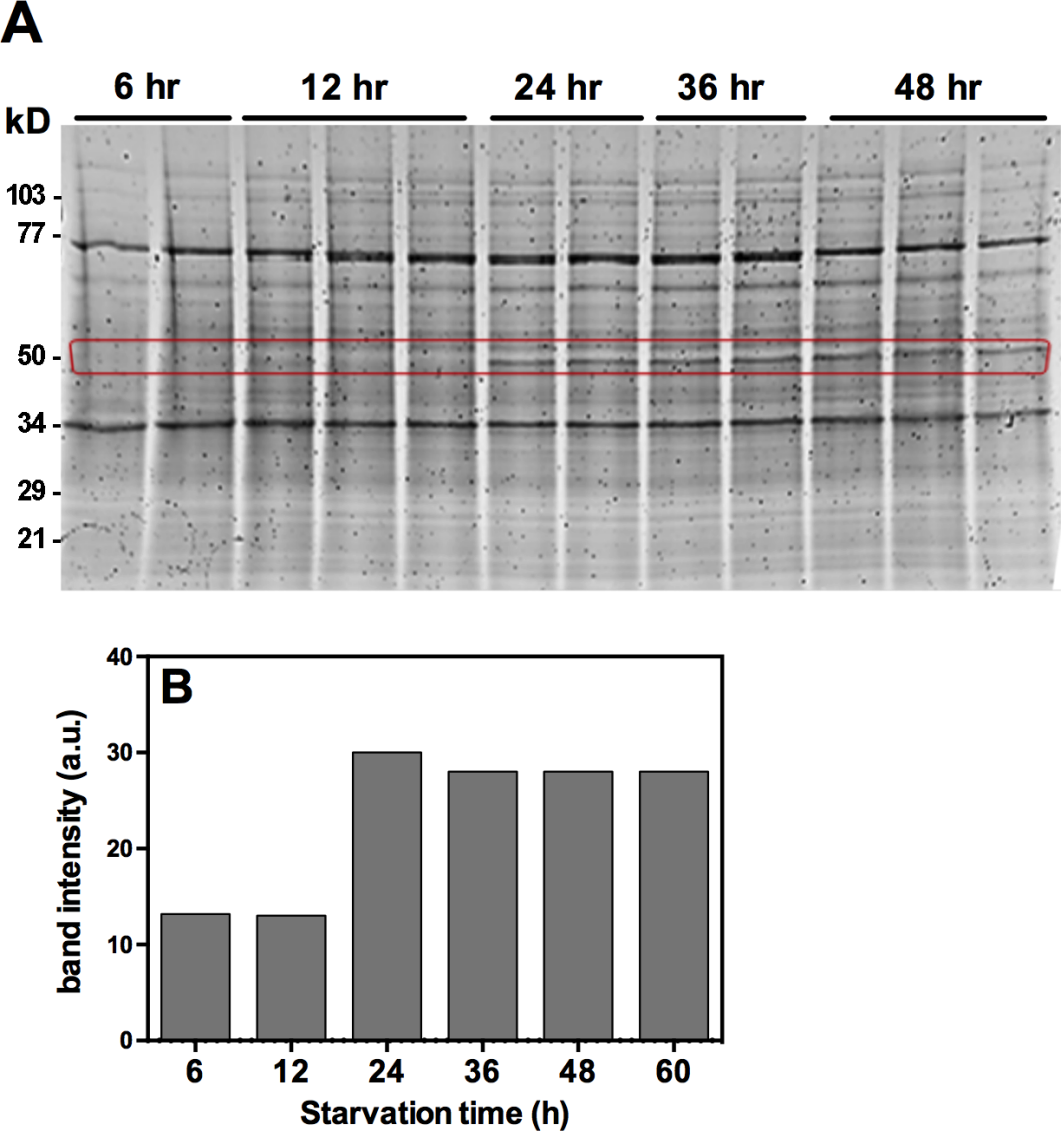
Effect of sulfur starvation on protein expression of *A*. *flavipes*. Spore suspension of *A*. *flavipes* was starved of sulfur for 6, 12, 24, 36, 48 and 60 h, and then transferred to media containing 5 mM L-methionine as the *S* source. (A) SDS-PAGE profile of total intracellular protein from these cultures. (B) Intensity quantification of a protein band of approximately 50 kDa (red rectangle) shows progressive increase of this band by 24 h and later time points.

Visual observation of the stained gel, with each lane loaded with equal amount (20 μg) of total protein, revealed that a band of approximately 50 kDa size was strongly induced by sulfur starvation at 24 h and later time points of *S* starvation (Fig 7A). The expression of this protein band was undetectable at the 6 h and 12 h intervals. Quantification of the intensity of this band revealed approximately 3-fold increase at the 24 h and later time points over the 6 and 12 h time points (Fig 7B). Interestingly, this 50 kDa protein under denaturing PAGE for *A*. *flavipes* is approximately of similar size of several sulfur amino acids assimilating pyridoxal 5'-phosphate dependent enzymes [3–8].

### Proteomic analysis of *A*. *flavipes* in response to sulfur starvation

**Fig 8:**
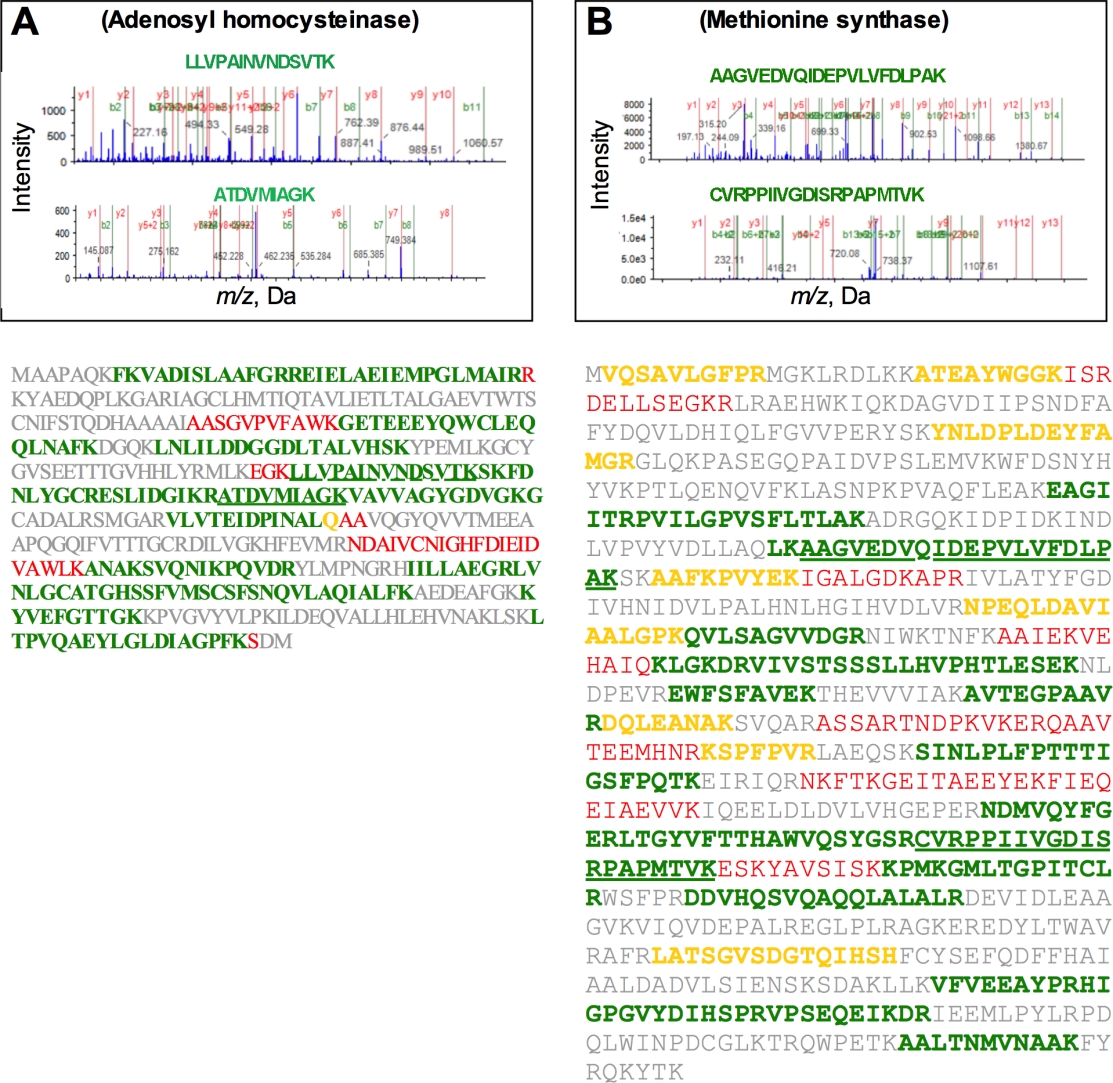
LC-MS/MS analyses of Adenosylhomocysteinase (A) and Methionine synthase (B) induced by *S* starvation. Upper panels show mass spectra of two representative peptides for each protein. Lower panels show amino acid sequence of *A*. *fumigatus* full length protein and its coverage by the mass spec peptides. Green colored fragments represent 100% identity of identified peptide with the annotated protein, whereas red, yellow and gray colors represent < 15, 30 and 50% similarity, respectively.

The *S* starvation-induced band (50 kDa Fig 7A) was subjected to proteomic analysis using LC-MS/MS. Since the *A*. *flavipes* genome is not available, we searched the identified peptides in the translated proteome of the closely related *A*. *fumigatus* genome and in the NCBI non-redundant protein database. These analyses revealed similarity to more than 100 proteins (S1–S4 Tables). Similarity and coverage of these peptides to the matching predicted proteins in the *A*. *fumigatus* genome are summarized in S1 Table and S2 Table. Among these proteins, methionine synthase, adenosyl homocysteinase, homocysteine synthase and ATP sulfurylase belonged to the *S* metabolism pathway. Two representative LC-MS/MS spectra of peptide fragments displaying 100% identity with the target proteins are shown in Fig 8. These proteins were well covered with high similarity by the identified peptides. Consistent with the PAGE gel data, the predicted molecular weight of most of these identified proteins ranged around 50 kDa. Full length amino acid sequence of *A*. *fumigatus* proteins that matched the identified peptide fragments with higher similarity are shown in Fig 8. These analyses suggest that some of these proteins could be overexpressed upon sulfur starvation. Gene ontology analyses of these proteins showed that most belonged to the catalytic and binding group in the molecular function category, to the metabolic, cellular and single-organism process in the biological processes category, and to intracellular cell functions (S1 Fig).

## Discussion

*A*. *flavipes* is considered an important source of sulfur amino acid metabolizing enzymes, some of which could be used for therapeutic purposes [4–8]. Various physiological conditions, abiotic stresses and toxic metals are suggested to affect sulfur metabolic pathways in fungi [45, 46]. Availability of sulfur amino acids have been reported to modulate tRNA thiolation and consequently protein expression [47]. Availability of sulfur nutrients have been suggested to affect cellular translational machinery, and rRNA and tRNA synthesis in fungi [48]. Similarly, sulfur availability and metabolism plays an important role in modulating the activity of enzymes in the sulfur metabolism pathways [4–8]. In this report, we investigated how *S* starvation and *S* sources affect activity and expression of key enzymes in sulfur metabolism in *A*. *flavipes*. In addition, we also conducted targeted proteomic profiling of *A*. *flavipes* in response to *S* starvation, which suggested that in addition to *S* metabolism other proteins involved in various cellular processes might also be affected by *S* starvation.

In our analyses, we found that *S* limitation affects various morphological and physiological characteristics of *A*. *flavipes*. Interestingly in our analyses, *S* starved cultures appeared whitish indicating a loss of pigment synthesis. Pigments in *Aspergillus* spp. are suggested to play a variety of roles in virulence and resistance to environmental stresses such as UV irradiation [49–51]. Loss of pigments in response to *S* starvation suggests that *S* limitation might adversely impact virulence and resistance to mutagens and other pigmentation-dependent processes in *A*. *flavipes*. It would be interesting to conduct detailed investigations of the role of *S* limitation in pigment synthesis using high throughput transcriptomic and pathological analyses in future studies.

Highest growth of the starved fungal spores was observed on MgSO_4_, followed by L-methionine, cysteine, cystine and glutathione. This could be due to higher sulfate transporter activity and/or accelerated incorporation into *S* containing molecules [15]. Consistent with the morphological observations, qPCR analysis also showed increased expression of sulfate permease. These observations are consistent with previous reports in *N*. *crassa* and *P*. *chrysogenum*, where sulfate permease I and II were highly expressed under sulfur limitation [17–19]. Similarly, L-methionine could also be transported by the same transporter that transports sulfate, albeit with different affinity [19], which could partially account for the higher fungal growth of *S* starved spores on these two sulfur sources. In presence of organic sulfur compounds, cells switch off ATP-sulfate transporters (sulfate permease) and switch on other transporting mechanisms [15]. Lower fungal growth observed on cysteine might be ascribed to complex transport mechanism of cysteine across the plasma membrane [52, 53]. Interestingly, intracellular pool of glutathione of sulfur starved *A*. *flavipes* was significantly more increased than non-starved controls on all tested sulfur sources (Fig 2). It could be argued that sulfur starvation expedited the assimilation of these compounds via activation of plasma membrane transporters partially resulting from their increased transcription, which would consequently enhance the flow of these compounds into the glutathione synthesis pathway [21].

Glutathione biogenesis, but not uptake, has been implicated in fungal virulence and pathogenesis, particularly in the context of sulfur and iron metabolism [27, 54, 55]. In our analysis, activity of glutathione reductase and glutathione peroxidase was increased in sulfur starved *A*. *flavipes* cultures than the non-starved ones when resupplied with MgSO_4_ and methionine. This finding is plausible, because negative effects of reactive oxygen species, which results from sulfur stress, could be cancelled by these enzymes via the glutathione oxidation and reduction acting as a cellular redox buffering [31, 56, 57]. However, the activity of glutathione *S*-transferase was similar under sulfur starving and non-starved cultures of *A*. *flavipes*, which is consistent with previous results [13, 58].

Protein profiling of *A*. *flavipes* displayed pronounced fluctuations in response to *S* starvation and culture on different *S* sources. This suggested that *A*. *flavipes* adjusts its protein expression and cell processes to adapt to the existing nutritional status. These observations are consistent with previous proteomic profiling studies, which reported substantial shift in proteome in response to various nutrient limitations in several fungi [25, 27, 44, 59–61]. These shifts in protein profiles are likely to fulfill the need for specific enzymes and other proteins that are specific for a particular nutritional limitation.

In this report, the activity and expression of key enzymes in sulfur assimilation and metabolism such as ATP- sulfurylase, sulfite reductase, cysteine synthase, cystathionine β-lyase (CBL), cystathionine γ-lyase (CGL), methionine γ-lyase, glutathione reductase and glutathione peroxidase were increased in *S* starved cultures of *A*. *flavipes* than the non-starved control, when resupplied with various sulfur sources (Figs 5 and 6). The increased activity could have resulted from the enhanced transcription or expression of these enzymes. Enhanced activity could be partially because of higher activity of sulfur transcriptional activator *metR*, which also displayed higher expression under the *S* starvation conditions. This is consistent with previous reports showing that metR enhances transcription of sulfur metabolism genes encoding sulfate permease, methionine permease, sulfite reductase, ATP-sulfurylase, homocysteine synthase and cysteine synthase [19, 22, 24].

Expression of cysteine synthase was increased in starved cultures compared to non-starved cultures. Cysteine synthase displayed higher activity and expression when grown on MgSO_4_ than on other S compounds (Fig 5), probably because of the direct incorporation of *O*-acetylserine into sulfide (Fig 4) [62]. The expression of L-methioninase, CBL and CGL was slightly increased in sulfur starved cultures of *A*. *flavipes* than in non-starved, which is consistent with our previous studies [6, 7, 13] and with the increased expression of *N*. *crassa* CGL under sulfur starvation [63]. The higher induction of CGL in *S* starved cultures when resupplied with L-methionine is reasonable, because CGL is a key enzyme in the trans-sulfuration pathway converting homocysteine to cystathionine [13].

Protein expression kinetics of *A*. *flavipes* using L-methionine sulfur source [64] showed a strongly induced band of 50 kDa size range. Proteomic analyses of this band showed similarity to several proteins. Some of these proteins are understandably associated with *S* metabolism, but many other proteins are not (S2 Table) suggesting that *S* metabolism affects other cellular processes. In future studies it would be interesting to investigate the role of some of these proteins using gene-knock-out and overexpression analyses.

## Conclusions

In conclusion, this study provides detailed analyses of responses of *A*. *flavipes* sulfur starvation at the physiological, enzymatic, gene expression and proteome levels. Our data show that sulfur starvation affects growth, nutrient uptake and expression of various enzymes related to sulfate transport, sulfur assimilation and metabolism. Our analyses suggest that *A*. *flavipes* adjusts its response to sulfur starving conditions by reprogramming its metabolic pathways to cope with the *S* limitation and *S*-induced stress. This study could provide information about how to enhance therapeutic uses of this fungus, especially its production of sulfur amino acid metabolizing enzymes and various antimicrobial compounds.

## Supporting Information

S1 Fig Gene ontology analyses of proteins induced by sulfur starvation. Gene ontology analyses were performed using the Blast2GO software.
(TIFF)

S1 Table Coverage and annotations of the identified protein in the predicted proteome of *Aspergillus fumigatus*. (XLSX)

S2 Table Molecular details and annotations of each identified peptides. (XLSX)

S3 Table Coverage and annotations of the identified protein in the NCBI non-redundent protein database. (XLSX)

S4 Table Molecular details and annotations of each identified peptides. (XLSX)
