# Supplementary figures and images for: Transcriptional and Proteomic Profiling of Aspergillus flavipes in Response to Sulfur Starvation
Source: PLoS One. 2015 Dec 3;10(12):e0144304. doi: 10.1371/journal.pone.0144304 (PMC4669086; doi:10.1371/journal.pone.0144304)

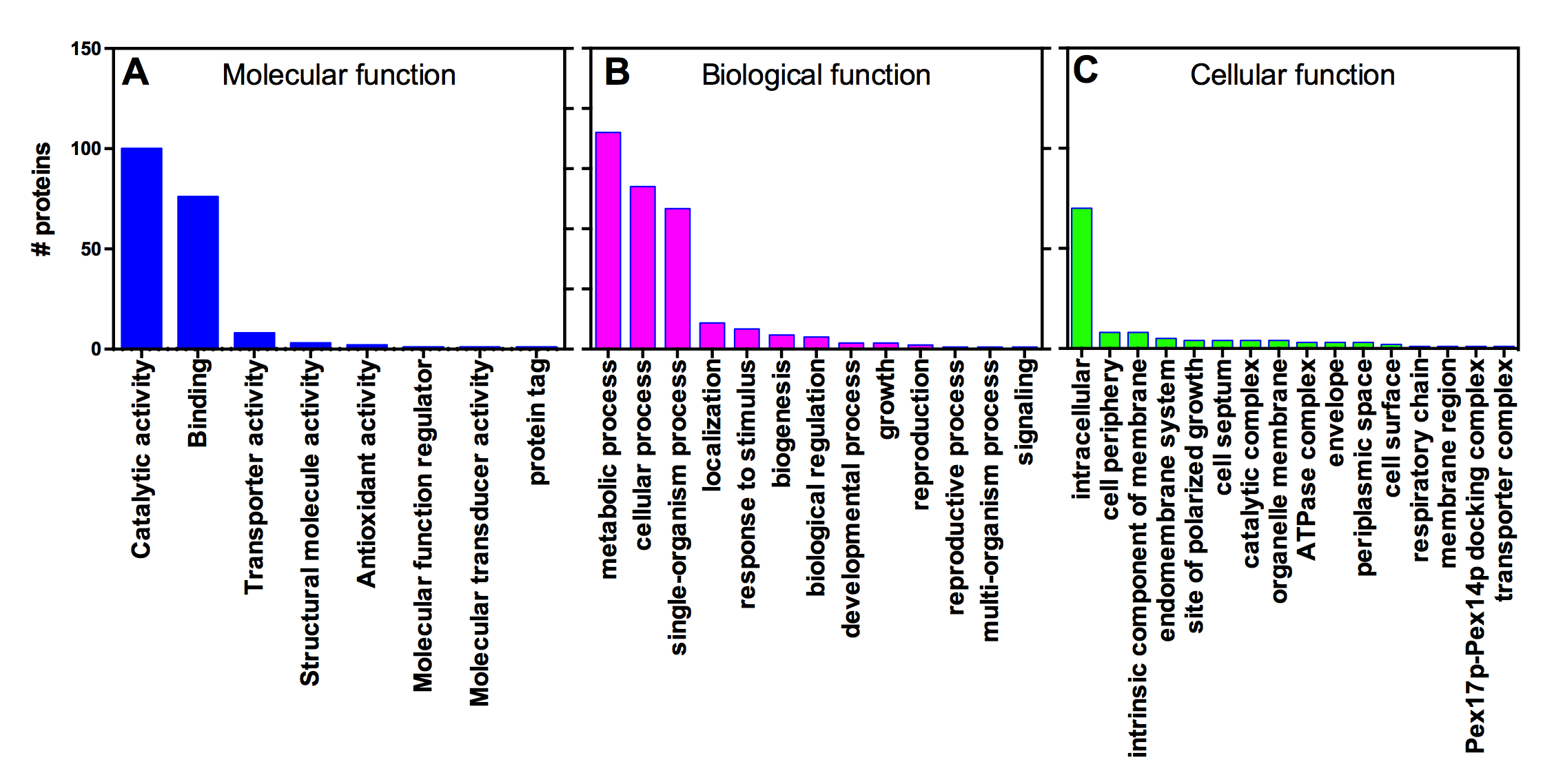

Supplement: S1 Fig — Gene ontology analyses were performed using the Blast2GO software. (TIFF) [file pone.0144304.s001.tiff]
